# Design and Preliminary Realization of a Screening and Early Warning Health Management System for Populations at High Risk for Depression

**DOI:** 10.3390/ijerph19063599

**Published:** 2022-03-18

**Authors:** Xin Chen, Liangwen Xu, Zhigeng Pan

**Affiliations:** 1 School of Public Health, Hangzhou Normal University, Hangzhou 311121, China; chenxinboss@stu.hznu.edu.cn; 2 Engineering Research Center of Mobile Health Management System, Ministry of Education, Hangzhou Normal University, Hangzhou 311121, China; 3 Institute of VR and Intelligent System, Hangzhou Normal University, Hangzhou 311121, China; 4 School of Artificial Intelligence, Nanjing University of Information Science & Technology, Nanjing 210044, China

**Keywords:** depression, high-risk population, screening and early warning, health management system, preventive treatment of disease, app, SMS platform

## Abstract

Depression has a high incidence in the world. Based on the concept of preventive treatment of disease of traditional Chinese medicine, timely screening and early warning of depression in populations at high risk for this condition can avoid, to a certain extent, the dysfunctions caused by depression. This work studied a method to collect information on depression, generate a database of depression features, design algorithms for screening populations at high risk for depression and creating an early warning model, develop an early warning short-message service (SMS) platform, and implement a scheme of depression screening and an early warning health management system. The implementation scheme included mobile application (app), cloud form, screening and early warning model, cloud platform, and computer software. Multiple modules jointly realized the screening, early warning, and management of the health functions of individuals at high risk for depression. At the same time, function modules such as mobile app and cloud form for collecting depression health information, early warning SMS platform, and health management software were designed, and the functions of the modules were preliminarily developed. Finally, the black-box test and white-box test were used to assess the system’s functions and ensure the reliability of the system. Through the integration of mobile app and computer software, this study preliminarily realized the screening and early warning health management of a population at high risk for depression.

## 1. Introduction

Depression is a chronic recurrent mental disease that causes great harm to human health [1]. Timely detection of populations at high risk for depression and targeted interventions and treatments can avoid the consequences of depression, such as suicide and other serious outcomes. The concept of “preventive treatment of disease” in traditional Chinese medicine for populations at high risk for depression involves screening and early warning before the disease becomes too severe and then taking appropriate intervention measures, to avoid severe and tragic consequences. Research on depression has an important role in guiding the formulation of public policies for the management of this disease. Hakulinen et al. [2] found that mental disorders, including depression, increase the risk of unemployment, that is, mental disorders may also cause indirect losses to individuals and families.

Therefore, it is of great significance to study the prevention and treatment of depression in high-risk groups. The key to a preventive treatment of a disease lies in how to give early warning on the risk of disease. Therefore, screening and early warning for populations at high risk for depression is the basis and key step for preventive treatment and management of depression. The depression screening scale Patient Health Questionnaire-9 (PHQ-9) is one of the effective screening tools for individuals at high risk for depression. PHQ-9 contains nine questions. The score of each question is 0–3, and the total score is 27. According to the PHQ-9 scoring standard, people with a score lower than 5 are healthy, whereas people with a score of 5 or more are defined as being at high risk for depression [3,4].

Timely early warning, early formulation of a health intervention plan, and actual health interventions are conducive to avoiding the tragic events caused by the intensification of depression and to allowing the recovery of mental health as soon as possible. Therefore, the development of a screening and early warning health management system for individual at high risk for depression has a good practical significance [5].

Aguilera et al. [6] used a machine learning algorithm in the mHealth app healthcare system for patients with depression and provided adaptive information to patients in relation to their behavior to increase the patients’ physical activity levels. Mobile health apps were used for self-care and health monitoring. Anderson et al. [7] analyzed how consumers use apps for health monitoring and associated benefits and put forward suggestions to improve these apps. Bae et al. [8] designed a user-centered web version of a depression symptom health management system in Korea, which mainly provides depression-related knowledge and a self-assessment scale without using the latest artificial intelligence technology. Deady et al. [1] used randomized controlled experiments to prove that using a specifically designed mobile app can reduce depressive symptoms and may promote mental health in the working population. The above research provided a reference for the design of this study. Our study focused on research on machine learning [6], screening and early warning models, and software design and app design methods [1,7]. The purpose was to study and design a screening and early warning health management system for populations at high risk for depression [8].

## 2. Materials and Methods

### 2.1. Health Information Collection Method

Depression health information can be collected by smartphone and mobile apps [9,10], smart watch, cloud form, etc. The traditional questionnaire method needs paper, printing, and a large number of interviewers; therefore, it requires time and expensive paper and manpower coordination and has a low efficacy. This study used cloud form and mobile app to replace traditional questionnaires in the collection of data. The cloud form was used to collect the data of the tested persons. The subjects were individuals interested in health management, including patients, doctors, and people who wished to check their health status. The purpose was to screen and warn individuals at high risk for depression.

Cloud form [11] is a kind of cloud service that can provide form-related services and applications. It is an innovative business of mobile Internet. Cloud form is often used for data collection. Using the form template, one can quickly create various data collection forms, such as in relation to a depression scale, depression health data (sleep habits, exercise level, diet, mood, etc.), demographic data, user satisfaction, activity registration information, etc. Cloud forms can be spread on social platforms through hyperlinks or QR codes, such as WeChat, QQ, WeChat group, QQ group, WeChat circle of friends, forums, etc., which can quickly catch the target population and accurately obtain the required data. It effectively improves the collection efficiency and reduces the collection cost. After a user fills the cloud form, the form data are saved in the cloud database. Cloud form users can access the cloud database through their own account to view or download cloud form data.

For complex data that cannot be represented by simple numerical values or words, such as voice data [12], video data, eye movement data [13], etc., a mobile app can be used to facilitate data collection. Due to the high popularity of smartphones, smartphones can be turned into data acquisition terminals by installing the designed app with data acquisition functions. At present, efficient and fast mobile app development platforms include App Inventor, APICloud, etc.

App Inventor is an app programming the environment that can be developed online. It can realize various complex functions through code block combination. It was designed and completed by Google engineers, Google enthusiasts, and users. APICloud [14] is a mobile app development platform launched by Youzi Technology (Beijing, China), a Chinese enterprise. The platform can simultaneously generate Android applications, apple IOS applications, wechat applets, and other applications with a set of codes and has been successfully applied in many large enterprises in China.

With the popularity of smart wearable devices, the sales of smart watches is gradually increasing. At present, the mainstream smart watches in the market generally integrate health management functions, such as OPPO smart watches, Apple smart watches, Samsung smart watches, and Huawei smart watches. The OPPO watch refined steel edition [15] integrates the function of exercise health partner, which can monitor exercise, heart rate, and sleep in real time. The OPPO watch refined steel edition can record detailed sleep data, including total sleep time, deep sleep time, and shallow sleep time, and can generate a detailed sleep data map. This is very convenient when collecting depression-related data. Collected health data can be submitted through the cloud form.

### 2.2. Generation Method of a Feature Database

Two types of data can be processed: simple data and composite data. Simple data are collected into a spreadsheet as required and generally include numerical data, text data, grade data, etc. The depression scale data, health data, and demographic data collected by a cloud table are simple data.

Simple data can be directly used in machine learning for their final representation. Simple data can be downloaded and extracted from the cloud database or directly entered into a spreadsheet. After simple data extraction, the original data are saved in an Excel spreadsheet. The Statistical Product and Service Solutions (SPSS) software can be imported to generate .sav data files for statistical analysis. It can also be saved in the SQL Server database to design a health management system.

Composite data, which gather a variety of feature information, cannot be directly modeled by machine learning [16]. They include voice data and video data. It is necessary to extract data features first. Taking voice data as an example, a recording file can extract 88 dimensional voice features (eGeMAPs, the extended Geneva minimalistic acoustic parameter set) [17] and generate a CSV file to save them. The extraction of speech features can be realized by Python programming. When each tester has only one recording file, the recording files of N individuals can generate N CSV files. If the value of N is small, the recording feature files can be merged manually to generate a total feature file database. When the N value is large, the code needs to be designed to automatically generate the feature database. Sometimes a tester has M voice samples, and the generation of the database will be more complex, and N * M files need to be merged.

Finally, simple data features and composite data features are combined to generate a database containing all features for machine learning modeling. For the same user ID, the data can be merged by adding columns. The feature extraction of speech samples can be completed by Python programming, and the relevant features can be written into CSV files. Then, by adding the for-loop statement, the feature extraction of all speech samples can be completed. Through the file merging operation, the feature database required for modeling can be generated.

### 2.3. Algorithms for the Screening and Early Warning Model

**Figure 1:**
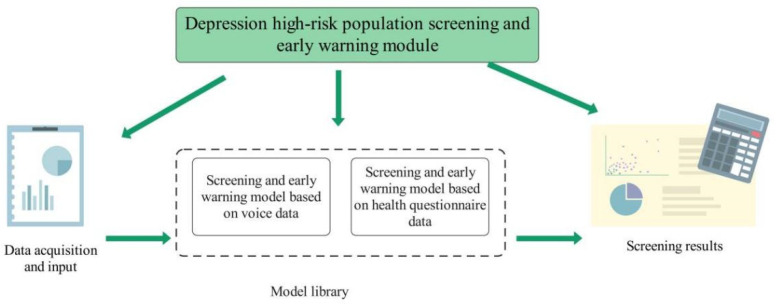
Scheme of the screening and early warning model.

After the construction of a feature database, in order to realize a system for the screening and early warning of populations at high risk for depression, we needed to design a machine learning screening and early warning model, which is shown in Figure 1. After the machine learning screening and early warning model was trained, the model could quickly analyze new test data and provide the screening results. The data in the feature database were randomly divided into a training set and a test set; the appropriate machine learning algorithm was selected for modeling, and the algorithm with good performance was selected. During training, if there are too many training features, it is necessary to filter them and select the most important ones before machine learning modeling. The commonly used methods are based on multiple the logistic regression algorithm, random forest algorithm, and multilayer perceptron neural network algorithm. Then the machine learning algorithm was used to establish the screening and early warning model for populations at high risk for depression. By comparing the performance indexes of various models, the optimal model was selected.

The algorithms used for machine learning modeling mainly include the decision tree algorithm, random forest algorithm, multiple logistic regression algorithm, support vector machine algorithm [18], multilayer perceptron neural network algorithm [19]. Taking the multilayer perceptron neural network as an example, the principle of machine learning for screening classification is introduced below.

**Figure 2:**
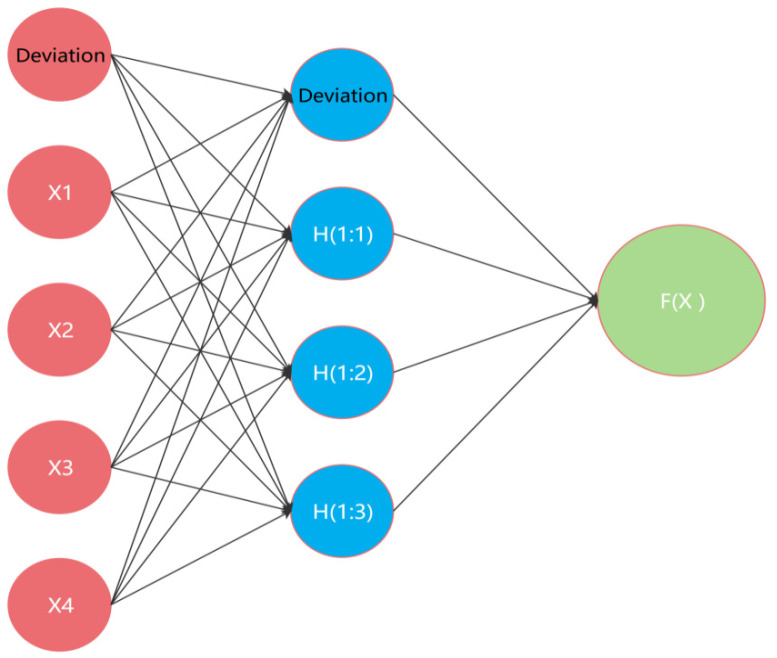
Network structure of multilayer perceptron.

Neural network is a very flexible adaptive learning system. It can automatically find patterns according to the input data, so as to make reliable predictions. Multilayer Perceptron (MLP) [20,21] is a powerful extension of perceptron and has the ability to approximate the nonlinear relationship between input layer and output layer with arbitrary accuracy. MLP [22] is a feedforward supervised structure, as shown in Figure 2. In addition to the input–output layer, it can contain multiple hidden layers and one or more dependent variables.

The dependent variables of MLP can be continuous, categorical, or a combination of them. If the dependent variables are categorized data, then MLP divides the records into the most appropriate categories based on the input data.

The red circles correspond to the input layer, and the independent variable X can be one or more. The blue circles are the hidden layer, which can include one or more hidden layers. The green circle is the output layer. The activation functions of a multilayer perceptron neural network are the Sigmoid function and the Tanh function. By using the activation function, nonlinear factors can be introduced to the neurons of the neural network, so that the multilayer perceptron neural network can be applied to more nonlinear models [22].

Sigmoid function:

(1) $$f(x)=\frac{1}{1+\exp(-x)}$$

The Sigmoid function has a value range of (0, 1), which is used for neuron output. It can map the output results to the interval of (0, 1), and can be used for binary classification.

Tanh function:

(2) $$f(x)=\frac{e^{x}-e^{-x}}{e^{x}+e^{-x}}$$

The value range of the Tanh function is (−1, 1). In the hidden layer, the Tanh function is better than the Sigmoid function.

### 2.4. Early-Warning SMS Platform

According to the results of the screening model for a population at high risk for depression, individuals with the predicted scale score belonging to this population are warned and then reminded through the SMS platform. Targeted intervention measures can be taken after early warning, such as medical scheme intelligent recommendations, online mental health interventions, virtual reality interventions, cognitive behavior interventions, traditional Chinese medicine exercise health interventions, etc., so to achieve preventive treatment and health management [23,24].

The warning signs are stored in the screening database of high-risk groups. The warning sign is set to 1 for individuals who need warning. In the warning database, the data table used for warning is named warningdatabase. The SQL code for filtering the warning groups is: select * from warningdatabase, where warning = 1.

The mobile phone numbers of all individuals who need to be alerted can be found in the warning data table, and then short messages with warning information can be sent in batches through the SMS platform. The SMS platform is a module of the health management system for the screening and early warning of populations at high risk for depression. Its purpose is to realize the early warning function. Early warning makes these individuals at high risk for depression understand their health status, which reflects the idea of preventive treatment of disease in traditional Chinese medicine. The methods for the development of the SMS platform mainly include the following two modes.

The first method is to integrate the existing mature platform system. Common SMS platform systems in China include the Tencent cloud SMS platform, the Feige SMS platform system, the China Mobile enterprise SMS delivery platform, etc. The SMS platform encapsulates the sending capability of SMS by an application programming interface (API). By calling the code, the SMS platform can be integrated in the health management system to realize the sending function of notification class and service class short message. It is also possible to operate directly on the SMS platform interface. Many large well-known domestic companies and units use third-party services to build SMS platform systems, such as People’s Insurance Company of China (PICC), Snow Beer, Hangzhou Normal University, etc. [25] This method has the advantages of fast deployment, powerful function, and cost saving.

The second way is to organize programmers to develop the platform according to the software development process and carry out requirements analysis, system design and development, system testing, software maintenance, and other processes. At the same time, the sending of SMS needs a cooperation with communication enterprises such as China Mobile, China Unicom, or China Telecom. The process is complex and requires large labor and time costs.

### 2.5. Overall System Design Scheme

**Figure 3:**
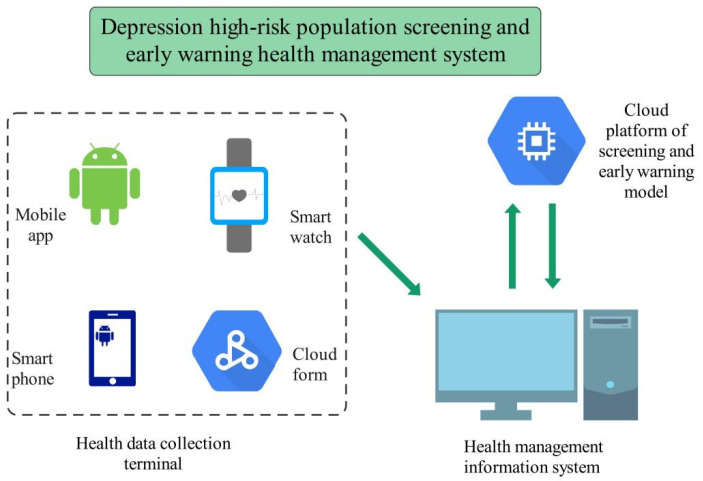
Design scheme of the screening and early warning health management system for populations at high risk for depression.

Based on the research of the above key technologies, it is of great practical value to design an application-oriented software system and promote its application in a university campus, so as to promote the mental health of college students and avoid the tragic consequences caused by the deterioration of depression, such as suicide. The system design scheme is shown in Figure 3 [10,26].

The screening and early warning health management system for populations at high risk for depression adopts three design schemes for integration: computer software, mobile app [27], and artificial intelligence-assisted diagnosis cloud platform.

High risk here refers to the high value in the PHQ-9 depression scale or other scales and the onset of depressive symptoms. For example, if PHQ-9 ≥ 5, depressive symptoms begin to appear. This can be predicted by a screening and early warning model using machine learning.

The health data acquisition terminal adopts four schemes: mobile app, smart watch, smartphone, and cloud form. The depression health management information system adopts the way of computer software. The depression screening and early warning model needs continuous model training and optimization. With the use of the system, new data are added, so the cloud platform is adopted. The cloud platform regularly provides the latest model download function. The screening and early warning of a population at high risk for depression is the key to the prevention and management of depression. Using a machine learning algorithm and the extracted modeling data features, a depression screening and early warning model can be designed. After cleaning and screening the collected feature data, they were randomly divided into 10 groups for training the model. A 10-fold cross validation was carried out to prevent over fitting of the model and increase its robustness. The programming and implementation of the above algorithm could adopt the following software: Python programming, Matlab programming, SPSS software, etc.

## 3. Results

### 3.1. Design of Information Collection App

**Figure 4:**
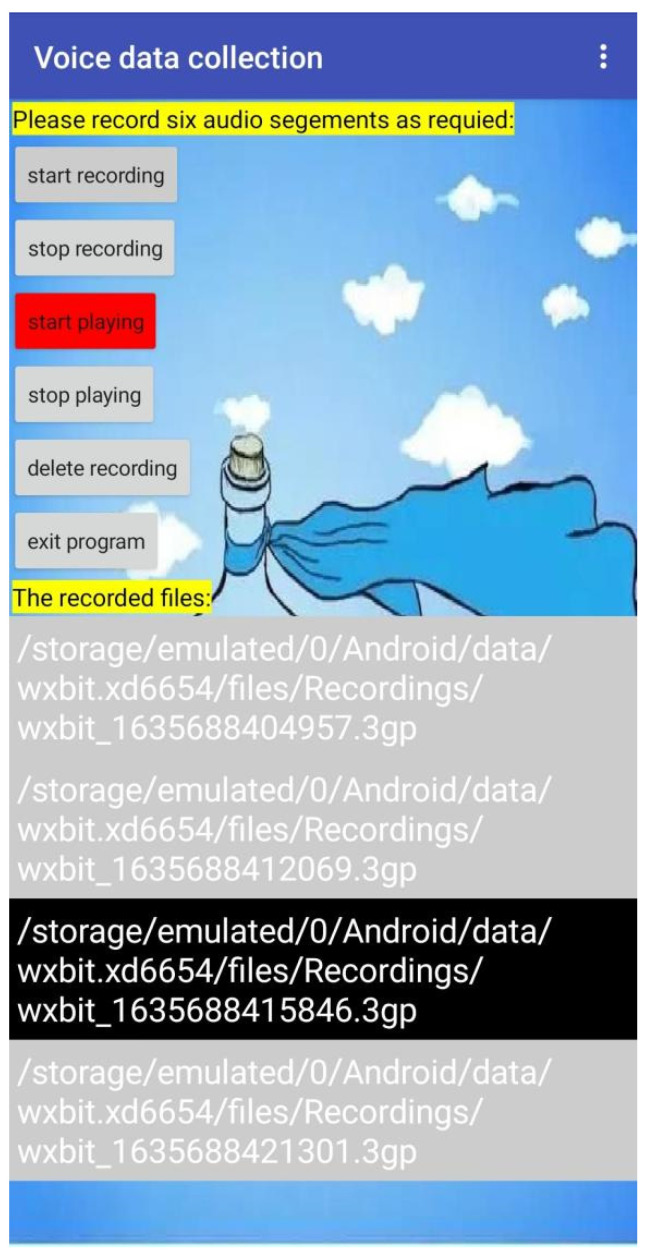
Voice data acquisition and management app.

Two ways could be used to collect health information in this system: one was to design a mobile app for health information collection, and the other was to collect questionnaire and scale data by cloud form. After installing an information acquisition app, the smartphone becomes a data acquisition intelligent terminal, which can save the cost of purchasing data acquisition equipment. In this study, a mobile app was mainly used to collect voice data. The development platform was Google’s App Inventor platform. The designed app operation interface is shown in Figure 4.

The App Inventor platform integrates a variety of objects and methods and uses object-oriented programming to quickly realize the required functions. First, a new project named “voice data collection” was created. Then, the component design and logic design of the project were carried out. In the component design module, two labels, six buttons, and one list display box were designed. The button functions included recording, playing, and deleting voice files, which were used for the management of voice files. The list display box displayed the storage location and file name of the recorded voice file and could perform file management operations such as audition and deletion by clicking the file name. Logic design realized the required functions by setting the properties and methods of various objects. For example, in regard to the logic design of the start playing button, when the button was clicked, the source file of the audio player object was set to the voice selected in the list display box, the background color of the button was set to red, and the start operation of the audio player object was executed [28].

After the component design and logic design were completed, the AI partner was installed on an Android mobile phone, and then the mobile phone could be used to test the project. Each control and function were tested item by item. In case of error or failure, the system was returned to be redesigned until the program design met the required function. Finally, app inventor was used to compile the project into Android Package (APK) files. After installing the project APK file on the mobile phone, the app was ready to run.

Cloud form was used to collect questionnaire and scale data. Cloud forms can automate various data collections in the cloud. The traditional paper questionnaire requires manual filling and then inputs the data into a computer for data analysis. It has a large workload, and it is easy to make mistakes. The cloud form can be filled directly online through a website or QR code. The data are saved in the cloud and can be analyzed directly, which can save paper and human and material resources. The commonly used cloud form systems in China include Questionnaire Star, Questionnaire Network, Jian Daoyun, Mike Form, etc.

**Figure 5:**
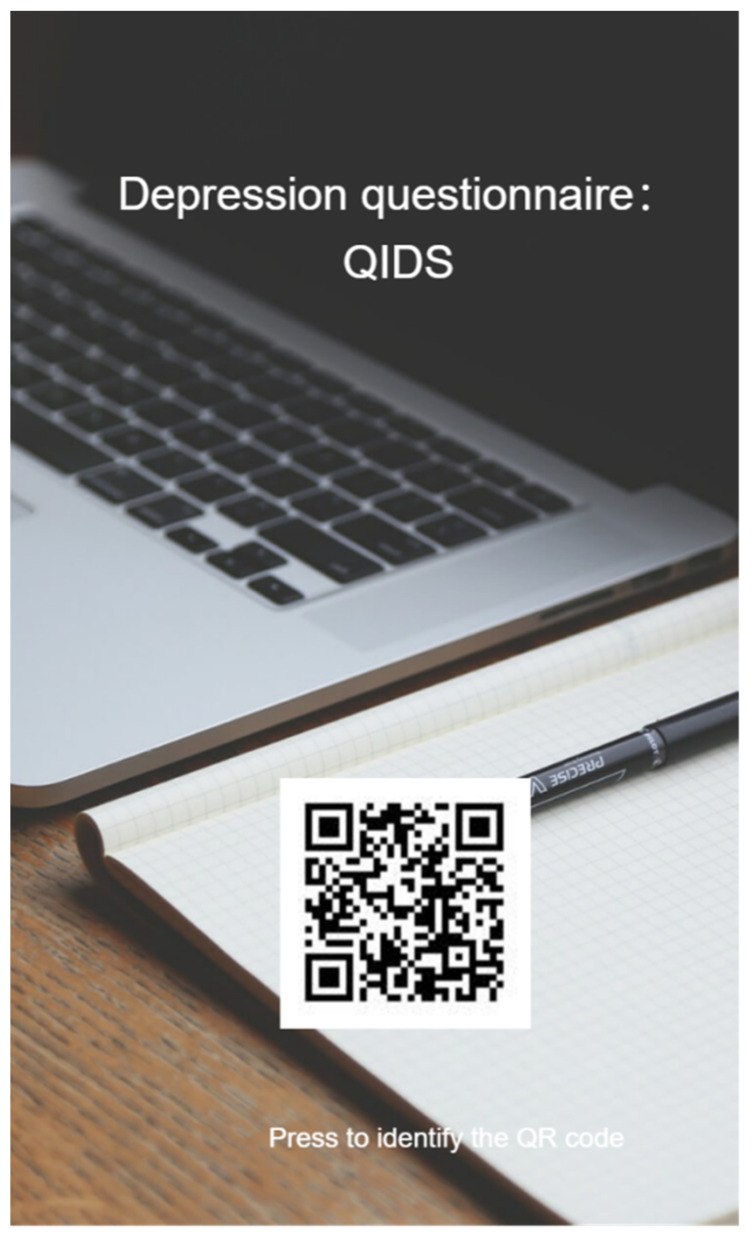
Example of the Questionnaire Star QR code poster.

In this study, Questionnaire Star was used to collect questionnaire and scale data. After the questionnaire was designed, QR codes or questionnaire links were sent using social tools such as WeChat and QQ, and questionnaire posters were made to collect data. The data were saved in the cloud, and the collected data could be downloaded and entered into the computer software for analysis. Or statistical analysis could be done directly in the cloud. The data acquisition poster is shown in Figure 5.

### 3.2. SMS Early Warning Function

**Figure 6:**
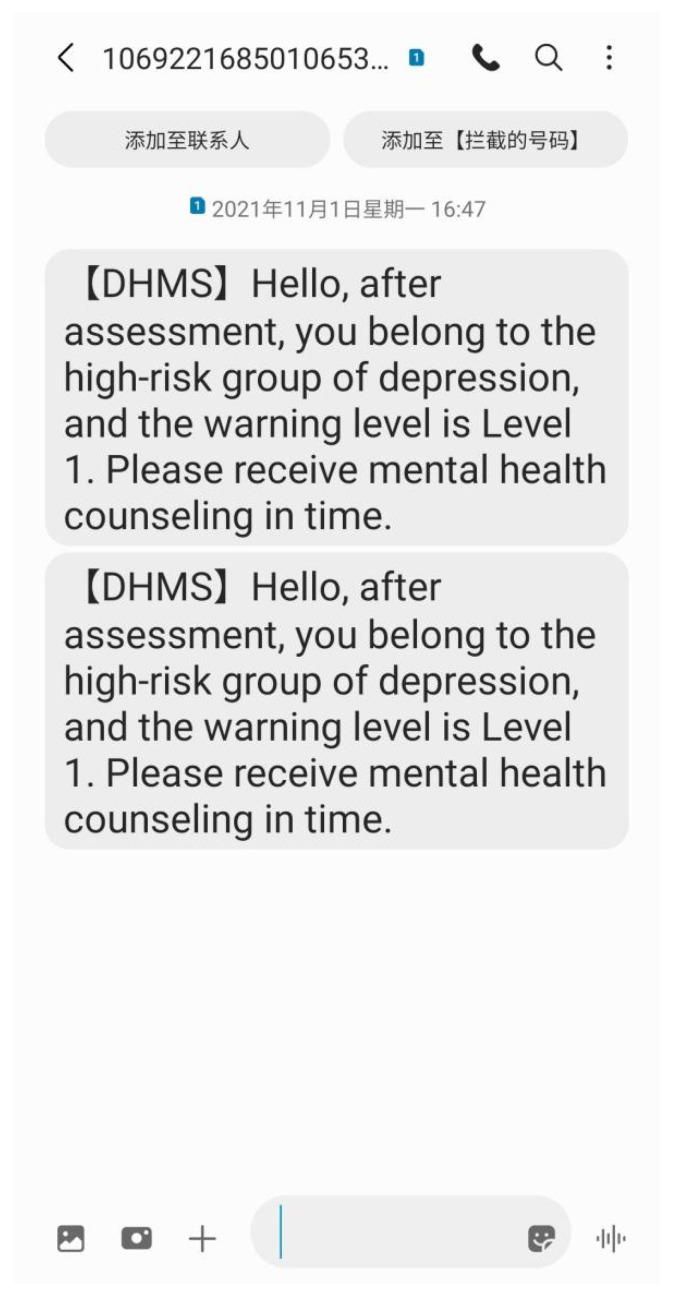
Warning information sent by the SMS platform.

After testing a population at high risk for depression using the screening and early warning model, the groups that need early warning are marked. Their warning value will be 1. TO query early warning database, the select SQL statement is used to filter the information for groups that needs early warning, including their mobile phone number, and then the SMS platform is alerted to activate the early warning operation [29]. Through the Application Programming Interface (API) provided by the third-party SMS platform, it is convenient to call the third-party SMS platform for the early warning of individuals at high risk for depression. The SMS platform has the functions of SMS delivery, SMS template, address book, SMS sending record, and so on. An example of SMS platform alert information is shown in Figure 6.

Figure 6 shows a health intervention message sent to customers by the SMS intervention platform. The mobile phone model receiving the SMS was Samsung Note20 Ultra 5G. The picture shows a screen capture of the mobile phone after receiving the SMS. DHMS refers to the system and is the acronym of “depression health management system”. For key groups, multiple text messages can be sent to attract attention.

### 3.3. Design Scheme of the Health Management System Module

The screening and early warning health management system for populations at high risk for depression mainly includes five functional modules: user management module, data collection module, screening and early warning model module, screening result database query module, and early warning short-message platform module.

The user management module can set the user account of the system and manage the account classification. Different types of accounts are given different user permissions. For example, the administrator account has the permissions of all modules of the system. Customer users can only query the screening result database and upload the collected data. The data acquisition module and the early-warning SMS platform module were described in detail.

In our early research, a decision tree model of screening and early warning based on voice data was designed and published in an SCI journal [30]. In this study, the decision tree model based on voice data was regarded as one of the possible models. After inputting original data, a model can automatically judge whether an individual belongs to the population at high risk for depression. The screening threshold for individuals at high risk for depression is different from that for individuals with depression. According to the data acquisition, the number of models can be expanded, e.g., by using a support vector machine (SVM) model based on voice and demographic data [4,31]. The screening and early warning model described here can be understood as a toolkit. In the screening result database query module, the SQL Server database 2019 was used to store the query data. The early warning data table was named warningdatabase. The warningdatabase includes relevant information of the testers, the predicted value of early warning information, and early warning signs. The SQL language can be used to query relevant information of all people who need early warning.

## 4. Discussion

The system adopted a modular design, and important functions were designed as independent modules. The design method has good portability and can be suitable for various complex platforms. At the same time, the system has good scalability, and functional modules can be added easily. In addition, by changing the training data of the screening model and retraining the screening model, the system can also be quickly developed into a screening early warning health management system for other diseases.

The screening and early warning health management system adopted two schemes for testing, i.e., white box test and black box test [32], to ensure the reliability of the system. The white box test is a code-based test that assesses the logical structure of all programs in the system. The white box test shows how the program works. By testing the writing, design, and branches of all programs one by one, if there are functions that do not meet the requirements, the code will be modified until all modules can run normally. Important modules were designed with a mature software platform to ensure the reliability of the system. The black box test is a function-based test. It does not consider the internal structure of the software. It regards the system as a black box and only considers whether the software function is realized. The black box test tested each functional module item by item and showed that all the modules could realize their functions.

According to the idea of preventive treatment of diseases in traditional Chinese medicine, the screening and early warning model focuse on the population at high risk for depression and automatically identifies the individuals who need early warning through the modeling algorithm. The SMS platform for real-time warning informs people at high risk for depression, who thus can understand and manage their health status. Then, it is suggested that interventions are taken to improve and promote the health of people at high risk for depression.

## 5. Conclusions

This research scheme integrated the theory of preventive treatment of traditional Chinese medicine and the theory of health management. Mobile app and cloud form were designed to collect depression health data. This method has advantages and is innovative with respect to traditional questionnaire collection methods. An algorithm was designed, and the automatic generation of a depression feature database was realized in Python language. A short message platform was used for the health early warning of individuals at high risk for depression. Traditional Chinese medicine recommends carrying out screening and early warning of people at risk for diseases. This method has been tested by the Chinese civilization for thousands of years. It is feasible and can be used as a practical policy in other countries.

The designed system integrates various functions, such as health information collection, health status machine learning screening, automatic early warning based on database technology, and a short-message platform [33], and preliminarily realized the preventive treatment and health management of a population at high risk for depression.

## Data Availability

Not applicable.
